# Effectiveness of bronchial thermoplasty for severe persistent bronchial asthma accompanied by *Pseudomonas aeruginosa* infection

**DOI:** 10.1016/j.rmcr.2022.101685

**Published:** 2022-06-13

**Authors:** Satoru Ishii, Motoyasu Iikura, Yuriko Sugiura, Rei Matsuki, Shinyu Izumi, Masayuki Hojo, Haruhito Sugiyama

**Affiliations:** Department of Respiratory Medicine, National Center for Global Health and Medicine, 1-21-1 Toyama Shinjuku-Ku, Tokyo, 162-8655, Japan

**Keywords:** BT, Severe asthma, *Pseudomonas aeruginosa*, Clarithromycin, %FEV_1.0_, AQLQ, AQLQ, asthma quality of life questionnaire, BT, Bronchial thermoplasty, CT, chest computed tomography, FeNO, Fractional exhaled nitric oxide, HRCT, High-resolution computed tomography, %FEV1, % post-bronchodilator forced expiratory volume in 1.0 s

## Abstract

Bronchial thermoplasty (BT) is a type of bronchoscopic treatment specifically used for patients with severe asthma. Most severe asthmatics receive systemic steroids and are at risk of being immunocompromised. This raises the clinical question of whether or not BT can be effectively and safely performed in such patients. Herein, we report a case highlighting the effectiveness and safety of BT in a patient with severe persistent bronchial asthma and *Pseudomonas aeruginosa* infection*.* We performed BT on a 46-year-old woman undergoing treatment for severe persistent asthma with inhaled steroids and 20 mg prednisolone orally. Although she was deemed to be infection-free before the procedure, culture of endobronchial secretions obtained during the first BT procedure grew *Pseudomonas aeruginosa*. After the first BT, she was given clarithromycin 400 mg orally daily. The amount of sputum decreased with each BT session, and sputum culture for *Pseudomonas aeruginosa* turned negative by the third BT session. Respiratory function tests showed 23.7% improvement in % post-bronchodilator forced expiratory volume in 1.0 s (%FEV_1.0_) and the asthma quality of life questionnaire (AQLQ) score increased by 2.41 points after the third BT. Bronchial wall thickness decreased and infiltrative shadows on CT disappeared after the three BT sessions, along with decrease in the amount of purulent sputum. Improvement in her asthma symptoms, after three BT sessions allowed decrease in the prednisolone dose.

We report the effectiveness of BT and infection control in a severe asthmatic with *Pseudomonas aeruginosa* infection.

## Highlights

• Bronchial thermoplasty (BT) is a type of bronchoscopic treatment specifically used for patients with severe asthma.
• There is a clinical question that whether we can perform the BT effectively and safely on immunocompromised patients.
• We report the effectiveness of BT and infection control in a severe asthmatic with *Pseudomonas aeruginosa* infection.

## 1 Introduction

Bronchial thermoplasty (BT) is a type of bronchoscopic treatment used to reduce the amount of smooth muscle in the bronchial wall in patients with severe asthma. Many studies have found that it helps improve symptoms in patients with severe asthma [1,2].

Asthma Intervention Research (AIR) 2 trial previously showed that BT improved asthma-related quality of life and reduced the frequency of severe exacerbations compared to a sham-controll group. However, one patient in the BT group needed hospitalization due to lower respiratory tract infection in this study [2].

Severe asthmatics usually receive systemic steroids, which might render them immunocompromised. Additionally, high-dose steroids and BT both have the potential to worsen the patient's infection status. We herein report the effectiveness of BT and infection control in a patient with severe asthma accompanied by *Pseudomonas aeruginosa* infection.

## 2 Case report

A 46-year-old woman, who was a non-smoker, was undergoing treatment for severe persistent asthma with inhaled steroids, long-acting β2 agonist, tiotropium inhalation, theophylline, montelukast, and prednisolone 20 mg orally. She was also on continuous omalizumab therapy that was prescribed at previous hospital.

Although the use of omalizumab seemed to improve the symptoms initially, they worsened again.

Prednisolone therapy was maintained because the symptoms worsened when the dose was reduced from 20 mg. Finally, the patient was referred to our hospital for BT.

Since her respiratory condition was poorly controlled despite intensive treatment.

At this time, her respiratory sounds were significant for wheezing in both lung fields.

Her vital parameters were: temperature: 36.2 °C, blood pressure: 116/70 mmHg, pulse rate: 82 beats/min with regular rhythm, respiratory rate: 16/min, and SpO₂ of 97% while breathing room air. Blood tests showed an IgE level of 15.3 U/ml, WBC count of 13400/μl with 0% eosinophils and CRP of 1.1 mg/dl (Table 1).

**Table 1:** Laboratory findings on admission.

＜Peripheral blood＞		＜Biochemistry＞		＜Serology＞	
WBC	13400/μl	TP	7.1 g/dl	Procalcitonin	0.04 ng/ml
Neu	93.0%	Alb	4.0 g/dl	IgE	15.3 U/ml
Lym	0%	AST	16 U/L	Cedar	3+
Mon	6.0%	ALT	14 U/L	Tick	2+
Eos	1.0%	LDH	221 U/L	House dust	1+
Bas	0%	BUN	13 mg/dl		
Hb	15.1 g/dl	Cr	0.5 mg/dl		
Ht	46.2%	CRP	1.1 mg/dl		
Plt	27.0 × 104/μl				

Respiratory function tests before BT showed a forced expiratory volume in 1 second of 2.05L (FEV_1.0_), %post-bronchodilator forced expiratory volume in 1 second (%FEV_1.0_) of 88% and a forced vital capacity (FVC) of 2.42L.

**Fig. 1:**
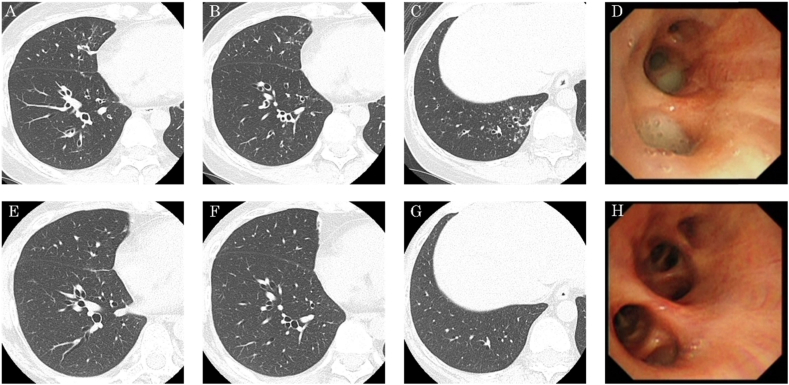
High-resolution computed tomography (HRCT) and bronchial thermoplasty (BT) findings; HRCT showed bronchial wall thickening and infiltrative shadows at the right inferior lobe before the first BT (A, B, C). Endobronchial inspection during the first BT procedure was significant for a large amount of yellow secretion in the right inferior lobe (D). HRCT performed after three BT sessions showed a decrease in bronchial wall thickness and disappearance of infiltrative shadows in the right inferior lobe (E, F, G). Endobronchial inspection during the third BT procedure did not show purulent sputum in the right inferior lobe (H). . (For interpretation of the references to colour in this figure legend, the reader is referred to the Web version of this article.)

CT showed bronchial wall thickening in all the bronchi and infiltrative shadows in the right lower lobe before the first BT (Fig. 1A, B, C). No bacteria were identified by microscopic evaluation of sputum that she spit herself at our out-patient department. Based on the inadequate asthma control despite various treatments, and lack of evidence of active infection at this stage from patient's general condition and blood sampling results, we decided to perform BT.

Investigations such as lung function tests, chest computed tomography (CT), and asthma quality of life questionnaire (AQLQ) score were performed approximately 2 weeks before the first BT session and again approximately 3 weeks after the third session. CT evaluation was performed using the same machine before and after treatment. Pre-bronchodilator values of pulmonary function parameters (FVC, FEV_1_, etc.) were used for correlation with data obtained from CT performed on the same day.

Thermoplasty was performed with the approval of the Ethics Committee of our hospital (NCGM-G-001801-00). An Olympus P290 bronchofiberscope (Olympus, Tokyo**,** Japan) with an Alair Bronchial Thermoplasty System (Boston Scientific Corporation, Tokyo, Japan) was used as the high-frequency energization system, CT was performed using an Aquilion ONE™ TSX-301A CT scanner (Toshiba Medal Systems, Tokyo, Japan), and pulmonary function tests were performed using computerized equipment (model CHESTAC-8100; CHEST MI, Inc., Tokyo, Japan).

The patient was given oral prednisolone at a dose of 50 mg for 3 days before the procedure, on the day of the procedure, and the day after the procedure, to minimize post-treatment airway inflammation. This resulted in disappearance of the wheezing at tKhe time of admission. The first thermoplasty session consisted of 32 activations in the right inferior lobe. Endobronchial inspection during the first BT procedure was significant for large amounts of yellow secretions in all bronchi, that grew *Pseudomonas aeruginosa* on culture (Fig. 1D). After confirming the absence of drug resistance in drug susceptibility tests, clarithromycin 400 mg daily was prescribed after the first BT.

The second bronchoscopic lumen evaluation showed a decrease in airway secretions.

**Fig. 2:**
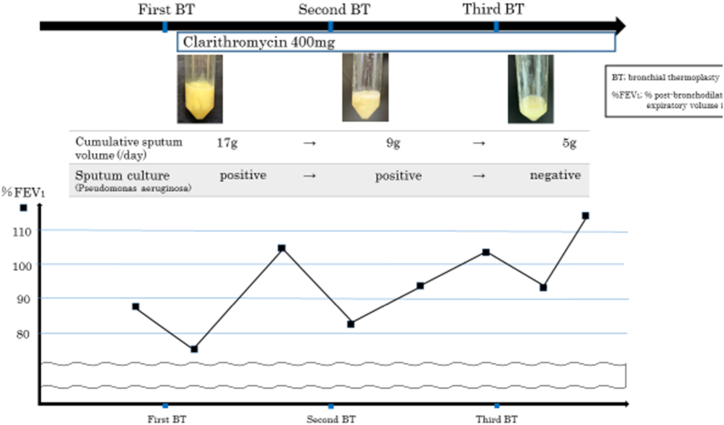
Time course of % post-bronchodilator forced expiratory volume in 1.0 s (%FEV1), cumulative sputum volume, results of sputum bacterial culture, and clarithromycin treatment; Pulmonary function improved after the third bronchial thermoplasty (BT) session. Cumulative sputum volume and results of sputum bacterial culture for *Pseudomonas aeruginosa* improved after the third BT session.

The second session consisted of 34 activations in the left inferior lobe, and the third session consisted of 56 activations in the left upper lobe, the lingular segment, and the right upper lobe. The amount of sputum in 24 hours was 17 g after the first BT session, decreasing to 9 g after the second session and 5 g after the third session (Fig. 2).

Sputum culture for *Pseudomonas aeruginosa* was positive at the first and second BT, but negative at the third BT session.

Respiratory function tests showed that %post-bronchodilator forced expiratory volume in 1 second (%FEV_1.0_) improved from 88% before treatment to 111.7% after the third BT. AQLQ score improved from 1.84 points before treatment to 4.25 points after the third BT.

Fractional exhaled nitric oxide (FeNO) did not change much, decreasing from 20 to 19 ppb, and eosinophil count and IgE levels remained unchanged.

CT showed a decrease in bronchial wall thickness in all bronchi, and disappearance of infiltrative shadows in the right inferior lobe after the three BT sessions (Fig. 1E, F, G). The amount of purulent sputum in all the bronchi decreased after the third BT session (Fig. 1H). Finally, since her asthma symptoms had improved, the prednisolone dose was decreased from 20 mg before the first BT session to 10 mg after the third session.

## 3 Discussion

To the best of our knowledge, the present case is the first report on the effectiveness of BT and infection control for severe persistent bronchial asthma accompanied by *Pseudomonas aeruginosa* infection. Research in Severe Asthma (RISA) study, in which severe asthmatics who were treated with BT were followed up for 1 year, showed improvements in the AQLQ score and preFEV_1.0_ with treatment [1]. In the AIR2 study, BT patients were followed for 5 years and were seen to have an improvement in AQLQ scores relative to those in the control group. Additionally, the proportion of patients with severe symptoms declined to 44%, and the number of emergency outpatient visits also decreased to 78% [2]. We also previously reported improvement in the patient's quality of life (QOL), with the decrease in exacerbations, symptoms, and lung function, following BT in Japanese asthmatic patients [3]. The main mechanism of action of BT is the reduction of airway smooth muscle. Similarly, in our case, CT showed dilation of the bronchial lumen and decreased bronchial wall thickness after BT.

Pretolani et al. reported that BT reduces airway smooth muscle area, subepithelial basement membrane thickness, nerve fibers and epithelial neuroendocrine cells in patholohical findings [4].

However, BT is considered invasive for the patient because it is performed three times using bronchoscopic maneuvers, and patients are given high doses of prednisolone before the BT. Further, care should be taken to prevent the occurrence or exacerbation of infection following BT. In the AIR2 trial, one patient in the BT group needed hospitalization due to respiratory tract infection after BT [2]. In our case, endobronchial inspection during the first BT procedure revealed the presence of a large amount of yellow secretions that grew *Pseudomonas aeruginosa* on culture.

Leukocytes increased due to the effect of oral steroids in blood sampling, and CRP did not show an increase as in the data of the previous hospital.

*Pseudomonas aeruginosa* infection was not judged as being active, based on the patient's general condition and blood sampling results. Regarding the usefulness of macrolide therapy for bronchial asthma, Amayasu et al. reported improvement in asthma symptoms, eosinophil count in blood and sputum, and airway hyperresponsiveness by administering clarithromycin to patients with allergic asthma [5]. Simpson et al. reported improvements in neutrophil counts and QOL with the addition of clarithromycin [6]. Clarithromycin has also been shown to be effective for airway hyperresponsiveness [7]. Tanabe et al. reported that clarithromycin suppresses IL-13 induced germ cell metaplasia [8]. In the present case, clarithromycin was administered for the purpose of suppressing germ cell metaplasia and reducing mucus secretion.

At our hospital, we have previous observed mucus hypersecretion and *Hemophilus influenza* in the first bronchoscopic sputum collection, we have experienced a case in which clarithromycin administration resulted in reduction in the amount of secretion [9].

In the present case as well, bronchoscopic evaluation of the airways for the second BT showed reduced airway secretions over both lung fields following treatment with clarithromycin. Sputum cultures were negative for *Pseudomonas aeruginosa* at the third BT, and there were no more infiltrative shadows on CT.

There are several limitations to the present report.

First, in this case, we were able to safety perform BT. However, since BT should not be performed in cases with active infection, the presence or absence of infection needs to be carefully evaluated. We have experienced cases in which *Hemophilus influenza* [9] and *Nocardia* spp [10] were observed in sputum culture when first BT was performed because it was judged that there no sign of infection before BT. Our experience suggests that even if sputum culture is negative in an outpatient setting, since bacteria may be found by bronchoscopic sputum collection, sufficient caution and appropriate measures are required when performing BT. Second, we previously experienced a case of Aspergillus infection attaching in the bronchi for which activation was performed by BT [10]. By BT procedure, ulcerous lesions were found in the bronchi and aspergillus fumigatus was detected with bronchial brushing. It should also be noted that BT might damage the airway epithelium.

Third, we used clarithromycin and her symptoms improved. But we don't know which was more beneficial than using antibiotics such as ceftazidime.

Fourth, in this case, although *Pseudomonas aeruginosa* disappeared in sputum culture within less than 3 months after BT, we should have ideally followed up the patient with sputum cultures for *Pseudomonas aeruginosa* for a longer time.

In conclusion, most severe asthmatics undergoing BT receive systemic steroids and are at risk of being immunocompromised. Even if they are asymptomatic, they may have endobronchial infections. Sufficient caution and appropriate measures are required when performing BT.

## Funding

Motoyasu Iikura received lecture fees from 10.13039/100016242Boston Scientific, Tokyo, Japan.

## Declaration of competing interest

The authors declare that they have no known competing financial interests or personal relationships that could have appeared to influence the work reported in this paper.

## References

[1] Pavord I.D., Cox G., Thomson N.C., Rubin A.S., Corris P.A., Niven R.M. (2007). Safety and efficacy of bronchial thermoplasty in symptomatic, severe asthma. Am. J. Respir. Crit. Care Med..

[2] Castro M., Rubin A.S., Laviolette M., Fiterman J., De Andrade Lima M., Shah P.L. (2010). Effectiveness and safety of bronchial thermoplasty in the treatment of severe asthma. Am. J. Respir. Crit. Care Med..

[3] Iikura M., Hojo M., Nagano N., Sakamoto K., Kobayashi K., Yamamoto S. (2018). Bronchial thermoplasty for severe uncontrolled asthma in Japan. Allergol. Int..

[4] Pretolani M., Bergqvist A., Thabut G., Dombret M.C., Knapp D., Hamidi F. (2017). Effectiveness of bronchial thermoplasty in patients with severe refractory asthma: clinical and histopathologic correlations. J. Allergy Clin. Immunol..

[5] Amayasu H., Yoshida S., Ebana S., Yamakoto Y., Nishikawa T., Shoji T. (2000). Clarithromycin suppresses bronchial hyperresponsinevess associated with eosinophilic inflammation in patients with asthma. Ann. Allergy Asthma Immunol..

[6] Simpson J.L., Powell H., Boyle M.J., Scott R.J., Gibson P.G. (2008). Clarithromycin targets neutrophilic airway inflammation in refractory asthma. Am. J. Respir. Crit. Care Med..

[7] Sutherland E.R., King T.S., Icitovic N., Ameredes B.T., Bleecker E., Boushey H.A. (2010). A trial of clarithromycin for the treatment of suboptimally controlled asthma. J. Allergy Clin. Immunol..

[8] Tanabe T., Kanoh S., Tsushima K., Yamazaki Y., Kubo K., Rubin B.K. (2011). Clarithromycin inhibits interleukin-13-induced goblet cell hyperplasia in human airway cells. Am. J. Respir. Cell Mol. Biol..

[9] Nagano N., Iikura M., Ito A., Miyawaki E., Hashimoto M., Sugiyama H. (2019). Bronchial thermoplasty for severe asthma with mucus hypersecretion. Intern. Med..

[10] Matsubayashi S., Iikura M., Numata T., Izumi S., Sugiyama H. (2018). A case of Aspergillus and Nocardia infections after bronchial thermoplasty. Respirol Case Rep.

