# Supplementary material for: Effectiveness of bronchial thermoplasty for severe persistent bronchial asthma accompanied by Pseudomonas aeruginosa infection
Source: Respir Med Case Rep. 2022 Jun 13;38:101685. doi: 10.1016/j.rmcr.2022.101685 (PMC9213246; doi:10.1016/j.rmcr.2022.101685)
Supplement: Multimedia component 1 [file mmc1.pdf]

FORTE

Forte Science Communications

Forte, Inc., KDX Shinjuku 286 Bldg. 5F, Shinjuku 2-8-6, Shinjuku-ku, Tokyo 160-0022, Japan
Tel: +81-3-3353-3545 Fax: +81-3-3354-3845 Email: info@forte-science.co.jp

April 14, 2022

To Whom It May Concern:

This is to confirm that Dr. Ishii's manuscript entitled "Effectiveness of bronchial thermoplasty for severe persistent bronchial asthma accompanied by *Pseudomonas aeruginosa* infection" has been edited by a native English speaker at Forte, Inc. Forte is a Japan-based company that employs editors and rewriters with a science background. We have been helping the Japanese scientific community to publish in international journals since 1987.

While this certificate confirms the authors have used Forte's editing services, we cannot guarantee that additional changes have not been made after our edits. Should you require any additional information about our services, please do not hesitate to contact our office.

Sincerely,

Forte, Inc.

株式会社フォルテ之印

KDX Shinjuku 286 Bldg. 5F
2-8-6 Shinjuku, Shinjuku-ku
Tokyo 160-0022, Japan
Tel: +81-3-3353-3545
Fax: +81-3-3354-3845
Email: info@forte-science.co.jp
